# Poly(Acrylonitrile–Butadiene–Styrene) as a Special β-Nucleating Agent on the Toughness of Isotactic Polypropylene

**DOI:** 10.3390/polym11111894

**Published:** 2019-11-16

**Authors:** Jingru Liu, Xinxu Zhu, Zheng Cao

**Affiliations:** Jiangsu Key Laboratory of Environmentally Friendly Polymeric Materials, School of Materials Science and Engineering, Jiangsu Collaborative Innovation Center of Photovoltaic Science and Engineering, Changzhou University, Changzhou 213164, China

**Keywords:** poly(acrylonitrile-butadiene-styrene), isotactic polypropylene, β nucleation efficiency, toughening, molding method

## Abstract

The influence of poly(acrylonitrile–butadiene–styrene) (ABS) as a special β-nucleating agent on the impact and tensile properties of isotactic polypropylene (iPP) were investigated by dynamic rheological measurements, wide-angle X-ray diffraction (WAXD), differential scanning calorimetry (DSC), scanning electron microscopy (SEM), and mechanical properties tests. It is found that the β nucleation efficiency of ABS is closely related to its concentration, dispersibility, and molding method for the iPP/ABS blends. The content of β-crystal (*K*_β_) rises with the incorporation of ABS and shows a maximum with the introduction of 1% ABS for compression-molded blends and 2% ABS for injection-molded blends, respectively, which is followed by a decrease in *K*_β_. The addition of a small amount of ABS has a significant reinforcing and toughening effect on iPP. Compared with the compression-molded samples, the ABS dispersed phase in injection-molded samples has a smaller particle size and a larger specific surface area, which are favorable for stress transmission and higher β nucleation efficiency, and therefore, better tensile and impact properties can be expected.

## 1. Introduction

Isotactic polypropylene (iPP), one kind of semi-crystalline polymer, owns several crystal forms, including monoclinic (α) [1], trigonal (β) [2], orthorhombic (γ) [3], and mesomorphic (smectic) [4] crystal forms. α-iPP is a conventional crystal form that spontaneously forms under conventional processing conditions. However, poor impact toughness and low heat distortion temperature limit its application in some areas. In contrast, β-iPP is metastable thermodynamically. It could be obtained under certain special conditions, including shear-induced crystallization, in a temperature gradient, and addition of a β-nucleating agent. Compared with other crystal forms, β-iPP owns a higher heat distortion temperature and impact toughness. Incorporating β-nucleating agent has been taken into account to be an effective way for the improvement of iPP toughness [5,6,7]. However, traditional β-nucleating agents belong to crystalline organic compounds, with the disadvantages of low thermal stability, high synthesis cost and color [8,9,10]. In contrast, polymers have advantages including nontoxicity, easy to process, and low price. The development of macromolecular β-nucleating agents has become a research hotspot in recent decades. It has been reported that thermoplastic phenolic resin [11], liquid crystal polymers [12,13,14,15,16,17,18], atactic polystyrene (aPS) [19], acrylonitrile–styrene copolymer (SAN) [19,20,21], and syndiotactic polystyrene [22,23] all have β nucleation activity. Although there is no general agreement about the β-nucleation mechanism of these macromolecular β-nucleating agents, it is believed that benzene rings existed in molecular structures play an important role, which may function as growth surface for the crystallization of β-iPP [19,20].

In general, the crystalline structure, crystal morphology, as well as crystallinity of crystalline polymers, have a remarkable influence on its properties. Moreover, its crystalline structure also depends on the molding method. For example, in the process of injection molding, both shear field and temperature field will cause the arrangement and stacking of iPP chains to be significantly different from the results of static crystallization [24,25,26]. In particular, for iPP with addition of macromolecular β-nucleating agents, the shear generated in the process of injection molding will also have an influence on the morphology and dispersibility of the macromolecular nucleating agent in iPP, thereby affecting its β nucleation efficiency [20]. Therefore, it is of great significance to study the impact of macromolecular β-nucleating agents on crystallization performance of iPP under different molding methods.

Poly(acrylonitrile–butadiene–styrene) (ABS) is a general thermoplastic. It combines the performance of the three components. Acrylonitrile makes the polymer resistant to chemical corrosion and improves the tensile strength and hardness; butadiene endows the polymer with excellent impact resistance; styrene has favorable high-temperature fluidity, and can improve the smoothness of the product. The above three components make ABS be a kind of plastic with good comprehensive performance. It is important to note that ABS has greater impact toughness and rigidity in contrast to iPP. Although many researchers have focused on the enhancement of compatibility between iPP and ABS [27,28,29,30,31,32,33,34,35,36], few reports are available on the influence of ABS on the crystallization of iPP [37]. In this paper, iPP/ABS blend specimens were prepared via compression molding and injection molding, respectively. The β nucleation activity, dispersibility and β nucleation efficiency of ABS were investigated. The effect of ABS on the impact and tensile performance of iPP was explored. Furthermore, the influence of the molding method on phase morphology, crystalline structure, as well as mechanical performance of iPP/ABS blends was also taken into account.

## 2. Materials and Methods

### 2.1. Materials

Isotactic polypropylene (iPP), trademarked as T30S, was purchased from Yangzi Petrochemical Company (Nanjing, China), with a melt flow rate of 3.2 g (10 min)^−1^ (230 °C, 2.16 kg). Poly(acrylonitrile–butadiene–styrene) (ABS), trademarked PA757, was supplied by Chimei Industrial Co. Ltd. (Taipei, Taiwan, China).

### 2.2. Sample Preparation

**Figure 1:**
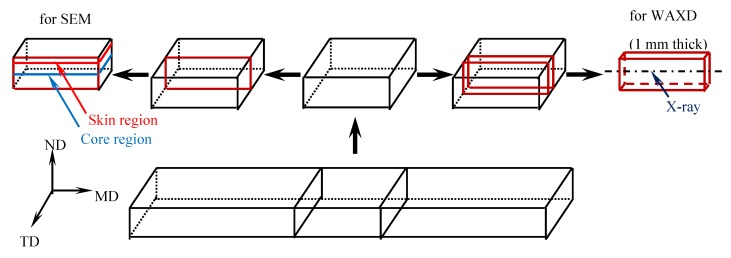
Schematic drawing of preparation of specimens for SEM and WAXD characterization: MD, the molded direction (i.e., flowing direction); TD, the transverse direction; ND, the direction normal to the MD-TD plane.

The ABS and iPP were melt blended by an internal mixer (SU-70C, Suyan technology Co. Ltd., Changzhou, China) at 180 °C for 7 min, with a rotation speed of 60 rpm. The proportions of ABS in the blends were 0, 0.3%, 0.7%, 1%, 2%, 4% by weight, respectively. For convenience, the blends were abbreviated as P-*x*A, where *x* indicates the mass fraction of ABS. The obtained blends were pulverized with a small high-speed pulverizer, and then two different molding methods were used to prepare the iPP/ABS blend specimens. (1) Compression molding. The blend powders were compression molded on a plate vulcanizing machine (406, Dongguan Xihua Testing Machines Co. Ltd., Dongguan, China) to form the tensile and impact specimens with a thickness of 2 mm and 4 mm, respectively. The compression molding conditions were set as follows: hot press at a temperature of 185 °C for 5 min under 7 MPa pressure, cold press for 1 min under 7 MPa pressure. (2) Injection molding. The blend powders were injection molded in a miniature injection molding instrument (HAAKE MiniJet II, Thermo Fisher, San Diego, USA) to produce the test specimens. The melting temperature was 200 °C, the mold temperature was 40 °C, the holding pressure was 0.6 MPa and the holding time was 10 s. The preparation of specimens for SEM and WAXD characterization is depicted in Figure 1.

### 2.3. Characterization

#### 2.3.1. Dynamic Rheological Properties

Dynamic rheological measurements were conducted in a rotational rheometer (MCR 102, Anton-Paar, Graz, Austria), using 25 mm parallel-plate fixtures with a constant gap setting of 0.9 mm. A nitrogen atmosphere was utilized to avoid thermal degradation. The storage modulus and complex viscosity were measured at 190 °C with respect to angular frequency, which was varied from 0.0398 to 628 rad/s in the linear viscoelastic region.

#### 2.3.2. Differential Scanning Calorimetry (DSC)

Samples of about 8 mg were analyzed under nitrogen atmosphere by a differential scanning calorimeter (DSC 6000, Perkin Elmer, Boston, USA). Each sample was first heated to 210 °C and maintained for 3 min to ensure an identical thermal history, following by cooling at a rate of 10 °C/min. As soon as the temperature reached 100 °C, it was reheated to 210 °C at the same rate. The reason for cooling to 100 °C is to avoid β-to-α recrystallization [38,39].

#### 2.3.3. Wide-Angle X-Ray Diffraction (WAXD)

The preparation of specimens for WAXD is illustrated in Figure 1. WAXD measurements of the core zone of the specimens were performed using a wide-angle X-ray diffractometer (D8 ADVANCE, BRUKER, Mannheim, Germany) with a Cu Ka radiation (*λ* = 1.54 Å). The data were collected from 5 to 35° (2*θ*) at a scan speed of 2°/min.

The relative content of β-crystal (*K*_β_) and total crystallinity (*X*_all_) were calculated by the following equations [40]:

(1) $$K_{\beta }=\frac{H_{\beta \left(300\right)}}{H_{\alpha \left(110\right)}+H_{\alpha \left(040\right)}+H_{\alpha \left(130\right)}+H_{\beta \left(300\right)}}$$

(2) $$X_{all}=1-\frac{A_{amorphous}}{\sum A_{amorphous}+A_{crystallization}}$$

where *H*_β(300)_ is the height of the β-form peak at 2*θ* = 15.9°, and *H*_α(110)_, *H*_α(040)_, and *H*_α(130)_ are the heights of three α-form peaks at 2*θ* = 14.1°, 16.9°, and 18.5°, respectively. *A*_amorphous_ and *A*_crystallization_ represent the areas of the scattering peak and diffraction peak, respectively.

The orientation parameters, including *A*_110_, *A*_130_, and *C*, representing the relative contents of the (110), (130), and (040) crystal planes, were calculated using Equations (3)–(5), respectively [41].

(3) $$A_{110}=\frac{H_{\alpha \left(110\right)}}{H_{\alpha \left(110\right)}+H_{\alpha \left(111\right)}+H_{\alpha \left(\bar{1}31\right)}}$$

(4) $$A_{130}=\frac{H_{\alpha \left(130\right)}}{H_{\alpha \left(130\right)}+H_{\alpha \left(111\right)}+H_{\alpha \left(\bar{1}31\right)}}$$

(5) $$C=\frac{H_{\alpha \left(040\right)}}{H_{\alpha \left(110\right)}+H_{\alpha \left(040\right)}+H_{\alpha \left(130\right)}}$$

where *H*_α(111)_ and *H*_α(-131)_ express the heights of α-form diffraction peaks at (111) and (-131) crystal planes, respectively.

#### 2.3.4. Scanning Electron Microscopy (SEM)

Microscopic observation was realized using a field emission scanning electron microscope (SUPRA 55, Zeiss, Karlsruhe, Germany) with an accelerating voltage of 5 kV. The samples were cryofractured parallel to the molding direction. All the specimens were gold sputtered before observation. Calculation of diameter of ABS dispersed phase was fully described in reference [42].

#### 2.3.5. Mechanical Properties Measurements

Tensile testing was performed using a universal testing machine (4302, Instron, Norfolk, USA) according to GB/T 1040-2006. The Izod notched impact strength was measured by using an impact tester (XJU-22, Chengde Testing Machine Co. Ltd., Chengde, China) according to GB/T 1843-2008.

## 3. Results and Discussion

### 3.1. Dynamic Rheological Behavior

**Figure 2:**
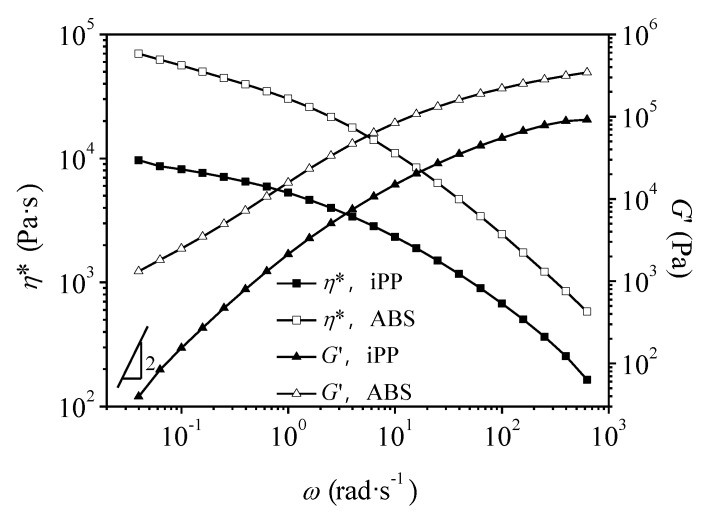
Storage modulus (*G*’) and complex viscosity (η^*^) with respect to angular frequency (*ω*) for pure iPP and ABS.

Figure 2 presents the results of storage modulus (*G*’) and complex viscosity (η*) obtained for pure iPP and ABS with respect to angular frequency (*ω*) at 190 °C. Obviously, for iPP, the slope of *G*’-*ω* curve at the low-frequency region is close to 2, exhibiting typical terminal behavior. However, ABS shows a deviation from the terminal behavior, indicating the presence of a longer relaxation time. This kind of non-terminal behavior could be attributed to the two-phase structure of ABS. In contrast to iPP, the used ABS has higher complex viscosity in the tested angular frequency range. This is beneficial to the dispersion of ABS in iPP matrix.

**Figure 3:**
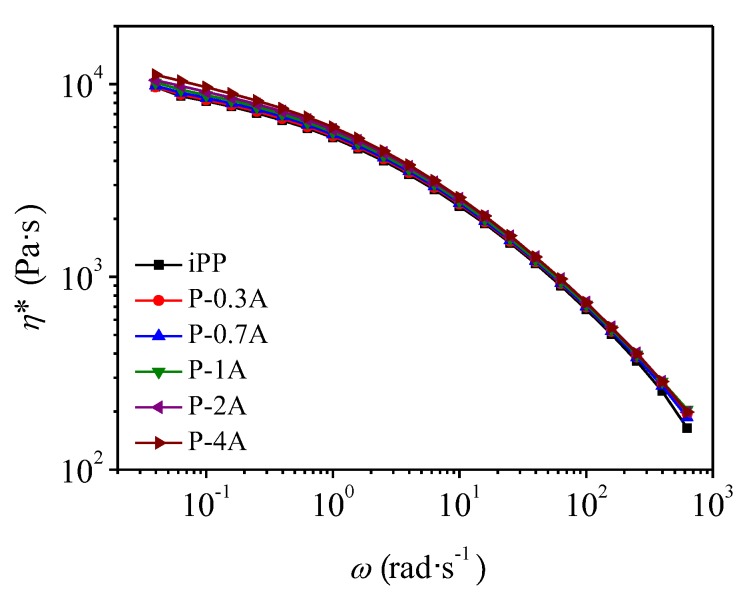
Complex viscosity as a function of angular frequency for iPP/ABS blends.

**Figure 4:**
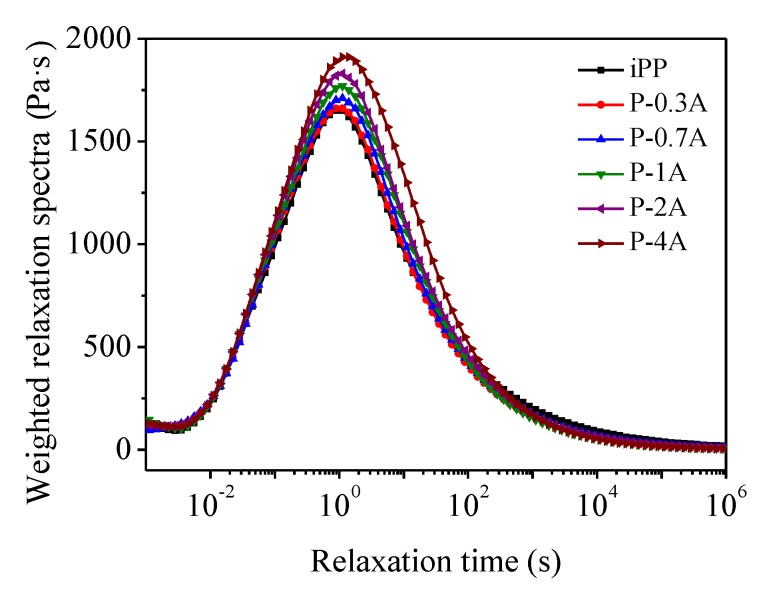
Weighted relaxation spectra of iPP/ABS blends at 190 °C.

Figure 3 shows the results of complex viscosity with respect to angular frequency for the blends. It could be seen that the complex viscosity of all the blends fell off with the increasing angular frequency, similar to that of pure components. In general, if the blend consists of two completely immiscible polymers with different viscosity, the viscosity of the blend should be lower than that of the two pure components due to the mutual repulsion of the macromolecular chains in the melts. However, the complex viscosity of the blends displayed an intermediate behavior between the pure components, implying that there was chain entanglement between iPP and ABS melts, which can be seen more clearly from the weighted relaxation spectra of the blends, as shown in Figure 4. With the increase in the ABS concentration, weighted relaxation spectrum presented an increasing trend in width, indicating more difficult relaxation for iPP resulting from the entanglement between iPP and ABS chains.

### 3.2. Crystallization and Melting Behavior

**Figure 5:**
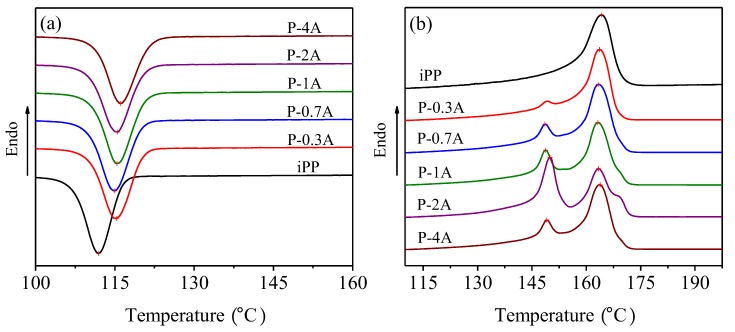
DSC curves of pure iPP and iPP/ABS blends on the first cooling (**a**) and the second heating scan (**b**).

Figure 5 presents the DSC curves of pure iPP and iPP/ABS blends on the first cooling (a) and the second heating scan (b), and the detailed crystallization and melting parameters are summarized in Table 1. It can be seen from the DSC curves that pure iPP shows one exothermic peak at 111.9 °C and one endothermic peak at 164.2 °C. For the iPP/ABS blends, the crystallization temperature of iPP increases from 111.9 to 116.1 °C with increasing ABS concentration on the first cooling scans, implying that ABS could perform the function of heterogeneous nucleation. On the second heating scans, the blends show two or three melting peaks. The first one appears at around 150 °C, corresponding to the melting of β-crystal of iPP, indicating that ABS owns a remarkable β nucleation activity on iPP. The second melting peak appears at around 163 °C, attributed to the melting peak of α-crystal for iPP. However, with a further increase in the ABS content, α’-crystal melting peak could be observed at around 169 °C as a result of melting and recrystallization of partial β-crystals. The crystallinity of the α-form (*X*_α_) and β-form (*X*_β_) and the relative content of β-crystal (*φ*_β_) are also listed in Table 1, which were calculated according to the standard procedures described in the literature [17]. Obviously, the blends reach the highest *X*_β_ and *φ*_β_ values with the addition of 2% ABS.

### 3.3. Crystalline Structure and Phase Morphology

**Figure 6:**
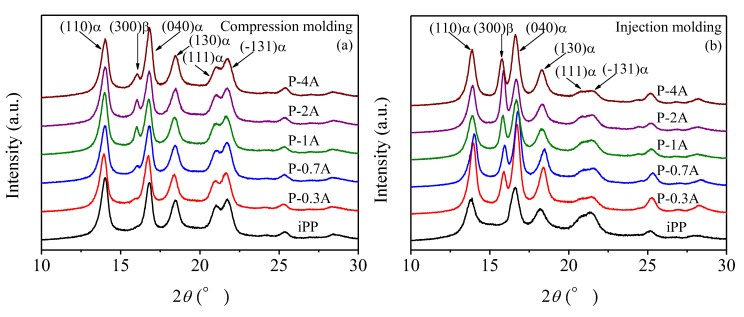
WAXD patterns of pure iPP and iPP/ABS blends prepared by compression molding (**a**) and injection molding (**b**).

**Figure 7:**
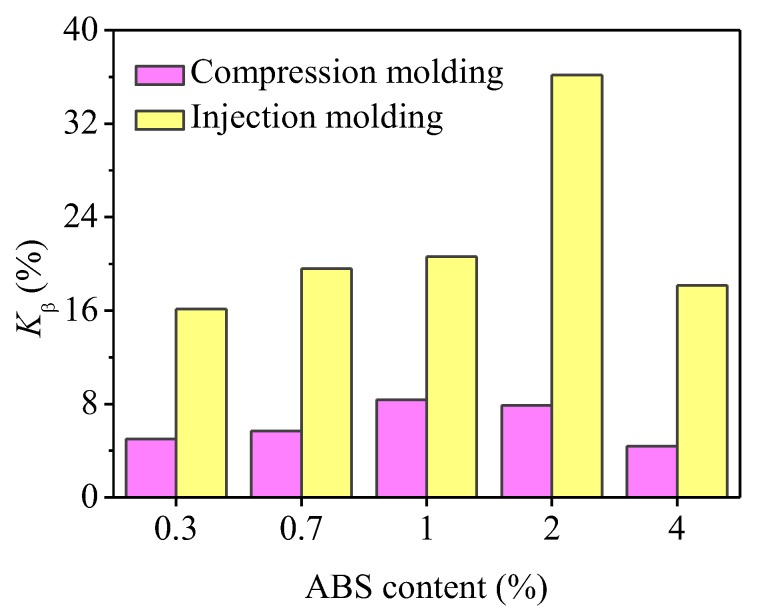
Relative content of β-crystal (*K*_β_) for the blends with respect to ABS content.

Figure 6a,b shows the WAXD patterns of pure iPP and iPP/ABS blends prepared by compression molding and injection molding, respectively. It can be found that only α-crystals exist in the pure iPP, and its characteristic peaks (2*θ*) are located at 14.1°, 16.9°, 18.5°, 21.1°, and 21.8°, corresponding to the (110), (040), (130) reflections and overlapping (111) and (-131) reflections, respectively. With introducing ABS into iPP, a new crystal structure, β-phase appears, with the characteristic peak (300) located at 15.9°. This is consistent with the DSC results. According to the Turner–Jones equation [40], *K*_β_ values calculated from the WAXD patterns in Figure 6, are displayed in Figure 7. It could be found that the *K*_β_ value increased with addition of ABS and reached a maximum when incorporating 1% ABS for compression-molded blends and 2% ABS for injection-molded blends, respectively. With a further increase in ABS content, a decrease in *K*_β_ could be found. The maximum *K*_β_ values of these two systems were 8.4% and 36.2%, respectively.

Generally speaking, the increase in the concentration of β-nucleating agent is in favor of the rise in the relative content of β-crystal. However, the results presented in Figure 7 indicate that the *K*_β_ values of iPP/ABS blends do not increase monotonously with respects to the ABS content. There are several different explanations for this phenomenon. Fujiyama et al. considered that when the content of β-nucleating agent exceeds a certain value, a large number of nuclei will cause the polypropylene chains to have difficulty in adjusting conformation to satisfy the chirality requirement of β-crystal, reducing the order of β-iPP in the direction of the C-axis, followed by a decrease in nucleation efficiency [41]. However, Su et al. found that further increase in the concentration of the nucleating agent deteriorated its dispersibility in the iPP matrix, leading to nonlinear change in *K*_β_ values [19,20].

Table 2 lists the corresponding WAXD parameters. Compared to the values of the orientation parameters, including *A*_110_, *A*_130_, and *C* for different blends, it could be found that with the increase in ABS concentration, all the parameters showed a trend of decreasing first and then rising. This is consistent with the results of styrene–butadiene–styrene block copolymer (SBS) induced crystallization in the iPP/SBS blends reported by Fujiyama et al. [43] He found that a further rise in content of β-nucleating agent will increase the ordering of α-iPP and reduce the ordering of β-iPP in C-axis direction, resulting in the difficult formation of β-crystal followed by a reduction in β-crystal content. A careful examination reveals that the iPP spherulite sizes, including *L*_110_ and *L*_040_, are barely affected by the introduction of ABS, indicating that the formed β-crystals did not have a significant influence on the size of α-crystals. In addition, as shown in Table 2, the iPP/ABS blends obtained by two different molding methods had higher values of crystallinity (*X*_c_) in contrast to pure iPP, owing to the heterogeneous nucleating effect of ABS. When the concentration of ABS exceeds a critical value, excessive amorphous ABS will hinder the crystallization of PP, followed by a decrease in crystallinity.

**Figure 8:**
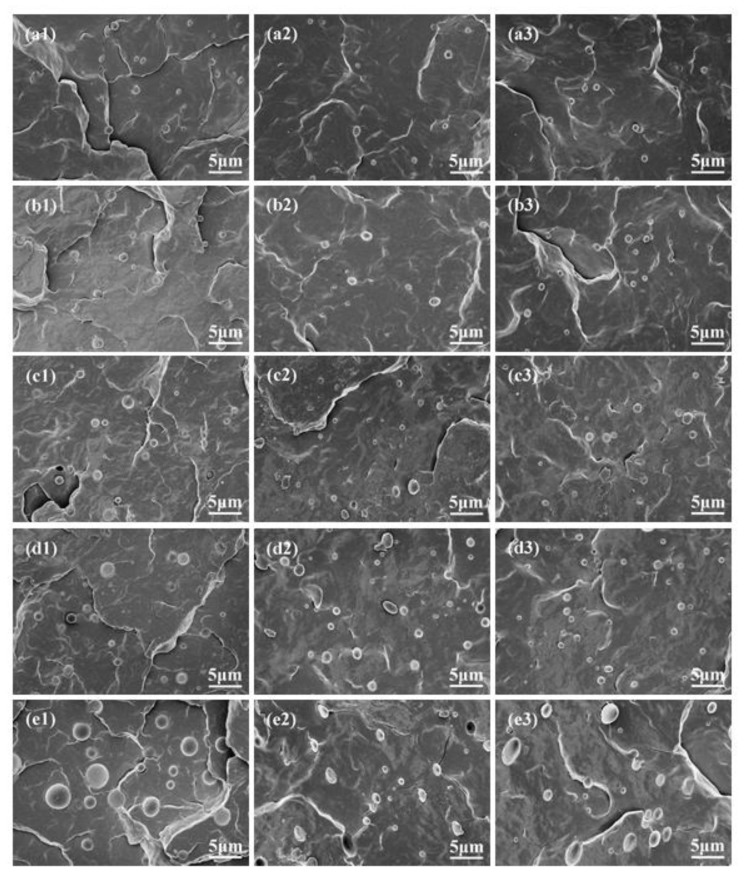
SEM micrographs of iPP/ABS blends. (**a**) P-0.3A, (**b**) P-0.7A, (**c**) P-1A, (**d**) P-2A, (**e**) P-4A. (**a1**–**e1**) compression-molded samples, (**a2**–**e2**) skin region of injection-molded specimens, (**a3**–**e3**) core region of injection-molded specimens.

To inspect the effect of ABS concentration on its morphology and dispersibility in the iPP/ABS blends prepared by compression molding and injection molding, SEM was used to observe the phase morphology, as displayed in Figure 8a1–e1 and Figure 8a2–e3, respectively. Especially, a skin region and a core region can form in the process of injection molding [20]. It is clear that ABS disperses as microspheres in the iPP matrix, indicating the immiscibility between the iPP and ABS. The size and polydispersity of ABS particles for different blends are listed in Table 3. Obviously, with the rise in ABS content, the size and polydispersity of ABS particles increased gradually. This could be attributed to the change of its dispersibility in the iPP matrix.

Compression molding is one of the traditional processing technologies and is mainly used for the production of thermosetting plastics. In the process of compression molding, pressure on plastics is relatively uniform, resulting in short flow distance and small deformation, and hence, a product with low internal stress will be obtained. The morphology investigation showed a spherical droplet structure of micron-scale for the ABS dispersed phase, as displayed in Figure 8a1–e1.

In the process of injection molding, the polymer melt is subjected to the dual effects of the temperature field and shear field, leading to its morphology being different from the result obtained under the compressing molding. In general, a typical skin–core structure exists along the thickness direction. It can be seen from Figure 8a2–e2,a3–e3 that, when the ABS mass fraction was lower than 2%, the ABS dispersed phase was microsphere-shaped in both the skin and core regions. These microspheres dispersed uniformly in the iPP matrix, and the size did not change significantly (about 1 μm), implying that the shear flowing has no influence on the morphology of the small-sized ABS microspheres in the process of injection molding. However, when the ABS content was up to 4%, the ABS particles in the skin and core regions were stretched into ellipsoids. In contrast to the ABS dispersed phase in the core region, the ABS in the skin region suffered more shear, resulting in smaller microspheres in the iPP matrix. Compared with compression molding, the ABS dispersed phase is more easily sheared in the process of injection molding. Therefore, the ABS microspheres showed a smaller size and polydispersity, regardless of the skin region or the core region, as listed in Table 3.

It is important to mention that shearing or stretching could also restrain the formation of β-iPP [24]. However, it can be found from Figure 6 that the shear effect has little impact on the crystal form of the used iPP. In contrast to the compression-molded iPP, the α-crystal diffraction peak intensity at (040) crystal plane for the iPP sample prepared by injection molding was significantly increased, indicating that the shear makes the iPP present preferential growth in (040) crystal plane. Furthermore, the *K*_β_ values for the blends prepared by injection molding were relatively higher, which is mainly due to the smaller size and better dispersibility of the ABS dispersed phase.

### 3.4. Mechanical Properties

**Figure 9:**
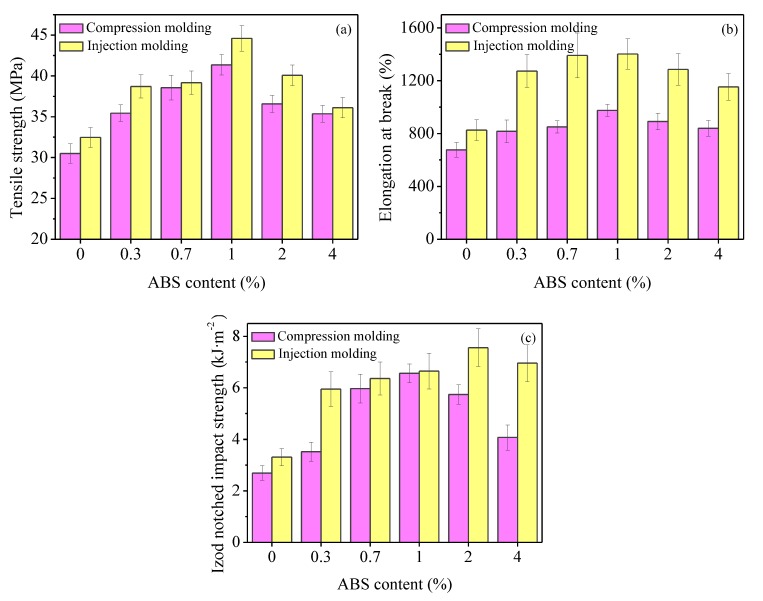
Effect of ABS content on (**a**) tensile strength, (**b**) elongation at break, and (**c**) impact strength of iPP/ABS blends.

**Figure 10:**
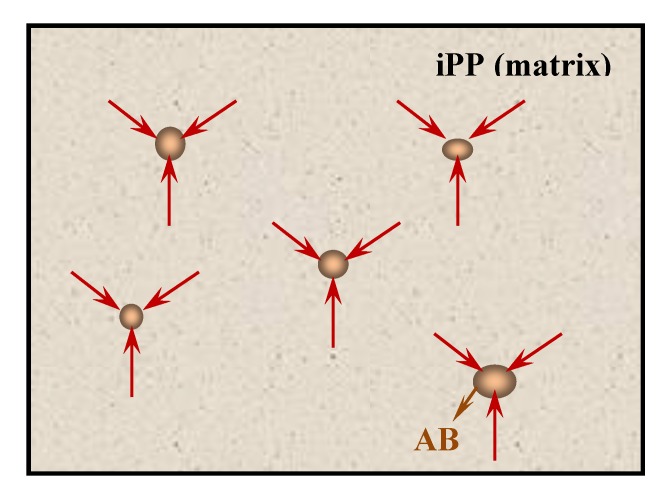
Diagrammatic drawing of a certain interfacial interaction between iPP and ABS when iPP is matrix.

Figure 9 presents the influence of ABS content on the tensile strength, elongation at break, and impact strength of the iPP/ABS blends. Obviously, incorporating a small amount of ABS leads to a remarkable improvement in both rigidity (tensile strength) and toughness (elongation at break, impact strength) of iPP. In contrast to pure iPP, the maximum values of tensile strength, elongation at break, and impact strength of the blends were 44.6 MPa, 1402.1%, and 7.6 kJ/m^2^, enhanced by 37.3%, 69.7%, and 128.4%, respectively. As mentioned earlier, there are certain chain entanglement and interfacial interaction between the iPP and ABS, as shown in Figure 10. During the stretching process, rigid ABS domains could play a role in stress transfer and reinforcement, and ultimately lead to higher tensile strength of the iPP/ABS blends in comparison with pure iPP. When the ABS content was 1%, the tensile strength reached its maximum. However, further increase in ABS content led to the decrease in the tensile strength. This could be explained by the interfacial debonding arising from the immiscibility between iPP and ABS.

When the sample is subjected to tensile or impact loading, debonding of the rigid organic particles of ABS occurs, followed by cavitation at the interface between the iPP and ABS. The cavitation and subsequent plastic deformation process could dissipate plenty of energy [42]. Moreover, ABS is an effective β-nucleating agent, and can induce the formation of β-crystal for iPP. Therefore, the iPP/ABS blends have superior fracture and impact toughness. ABS shows a remarkable β nucleation activity, which contributes to the improvement in iPP impact strength. It should be mentioned that a further increase in ABS content will reduce the order of β-iPP in the C-axis direction and deteriorate its dispersibility. Moreover, introducing excess ABS brings about the formation of defects in the blends. As a result, the values of elongation at break and impact strength fell off with a further increase in ABS content.

Compared with the compression-molded samples, the ABS dispersed phase in the injection-molded samples had a smaller particle size and a larger specific surface area, which is favorable for stress transmission and higher β nucleation efficiency, and therefore, better tensile and impact properties were obtained.

## 4. Conclusions

Poly(acrylonitrile–butadiene–styrene) (ABS) could generally serve as a modifier for isotactic polypropylene (iPP). In the traditional processing modification method, the iPP/ABS blends show unfavorable mechanical performance as a result of the incompatibility between ABS and iPP, as well as a high concentration of ABS. In this experiment, the mass fraction of ABS was controlled within 4%. The results show that a small amount of ABS can disperse uniformly in the iPP matrix. ABS shows a remarkable β nucleation activity on iPP. Its β nucleation efficiency is closely related to its concentration, dispersibility, and molding method for the blends. The *K*_β_ value increases by the introduction of ABS and reaches a maximum with the addition of 1% ABS for compression-molded blends and 2% ABS for injection-molded blends, respectively. The maximum *K*_β_ values of these two systems were 8.4% and 36.2%, respectively. Furthermore, there are certain chain entanglement and interfacial interaction between the iPP and ABS. During the stretching process, rigid ABS domains could play a role in stress transfer and reinforcement. As a result, the addition of a small amount of ABS has a significant reinforcing and toughening effect on iPP. In contrast to pure iPP, the maximum values of tensile strength, elongation at break, and impact strength of the blends were 44.6 MPa, 1402.1%, and 7.6 kJ/m^2^, enhanced by 37.3%, 69.7%, and 128.4%, respectively. Compared with the compression-molded samples, the ABS dispersed phase in injection-molded samples had a smaller particle size and a larger specific surface area, which is favorable for stress transmission and higher β nucleation efficiency, and therefore, better tensile and impact properties can be expected.

## Figures and Tables

**Table 1:** Crystallization and melting parameters of pure iPP and iPP/ABS blends.

Samples	First Cooling Scan		Second Heating Scan								
	*T*_c_ (°C)	Δ*H*_c_ (J·g^−1^)	*T*_m,β_ (°C)	△*H*_m.β_ (J·g^−1^)	*T*_m,α_ (°C)	Δ*H*_m.α_ (J·g^−1^)	Δ*H*_m_ (J·g^−1^)	*X*_β_ (%)	*X*_α_ (%)	*X*_all_ (%)	*φ*_β_ (%)
iPP	111.9	107.0	-	-	164.2	95.2	95.2	-	53.8	53.8	0
P-0.3A	115.3	100.5	149.3	7.3	163.6	89.6	96.9	4.3	50.6	54.9	7.9
P-0.7A	115.0	99.6	148.6	17.2	163.3	86.7	103.9	10.2	49.0	59.2	17.2
P-1A	115.4	99.7	148.7	26.4	163.2	72.7	99.0	15.6	41.1	56.7	27.6
P-2A	115.5	98.1	150.0	52.0	163.4	52.9	104.9	30.9	29.9	60.7	50.8
P-4A	116.1	97.4	149.1	21.4	163.8	80.0	101.3	12.7	45.2	57.9	21.9

**Table 2:** WAXD parameters obtained by two different molding methods.

Samples	Compression Molding						Injection Molding					
	*X*_c_ (%)	*A*_100_ (%)	*A*_130_ (%)	*C* (%)	*L*_110_ (nm)	*L*_040_ (nm)	*X*_c_ (%)	*A*_100_ (%)	*A*_130_ (%)	*C* (%)	*L*_110_ (nm)	*L*_040_ (nm)
iPP	60.6	51.2	34.9	37.5	16.1	16.0	53.9	84.3	69.1	45.0	16.0	15.5
P-0.3A	70.3	50.5	34.3	36.9	16.0	16.1	55.5	80.0	67.7	44.4	16.6	15.8
P-0.7A	72.3	50.1	33.4	36.5	15.6	15.3	61.4	70.4	57.9	43.1	16.1	15.9
P-1A	78.0	49.1	32.4	34.6	16.2	15.7	65.3	61.7	45.6	41.6	15.5	14.9
P-2A	75.1	52.2	35.5	35.3	15.5	16.0	73.6	70.9	52.3	37.8	16.1	14.5
P-4A	61.5	52.3	38.5	41.1	16.0	15.8	54.7	82.4	70.0	41.9	16.1	15.0

**Table 3:** The number-average diameter (*D*_n_), weight-average diameter (*D*_w_), volume-average diameter (*D*_v_), and polydispersity (PD) of ABS dispersed phase in iPP/ABS blends.

Blends	Compression Molding				Injection Molding							
					Skin Region				Core Region			
	*D*_n_ (μm)	*D*_W_ (μm)	*D*_V_ (μm)	PD	*D*_n_ (μm)	*D*_W_ (μm)	*D*_V_ (μm)	PD	*D*_n_ (μm)	*D*_W_ (μm)	*D*_V_ (μm)	PD
P-0.3A	1.109	1.149	1.215	1.096	0.863	0.899	0.901	1.044	0.882	0.918	0.922	1.045
P-0.7A	1.130	1.169	1.242	1.099	0.926	0.943	0.974	1.052	0.955	0.970	1.019	1.067
P-1A	1.149	1.234	1.385	1.205	0.979	1.097	1.069	1.092	1.055	1.128	1.161	1.100
P-2A	1.379	1.471	1.738	1.260	1.012	1.132	1.159	1.145	1.074	1.196	1.252	1.166
P-4A	2.376	2.689	3.169	1.334	1.528	1.419	1.815	1.188	1.567	1.769	1.995	1.273

## References

[1] Padden F.J., Keith H.D. (1959). Spherulitic crystallization in polypropylene. J. Appl. Phys..

[2] Turner-Jones A., Cobbold A.J. (1968). The β crystalline form of isotactic polypropylene. J. Polym. Sci. Part C Polym. Lett..

[3] Kardos J.L., Christiansen A.W., Baer E. (1966). Structure of pressure-crystallized polypropylene. J. Polym. Sci. Part B Polym. Phys..

[4] Piccarolo S., Saiu M., Brucato V., Titomanlio G. (1992). Crystallization of polymer melts under fast cooling. II. High-purity iPP. J. Appl. Polym. Sci..

[5] Fujiyama M. (1995). Structures and properties of injection moldings of β-crystal nucleator-added polypropylenes: Part 1 Effect of β-crystal nucleator content. Int. Polym. Proc..

[6] Varga J. (1992). Supermolecular structure of isotactic polypropylene. J. Mater. Sci..

[7] Luo F., Geng C., Wang K., Deng H., Chen F., Fu Q., Na B. (2009). New understanding in tuning toughness of β-polypropylene: The role of β-nucleated crystalline morphology. Macromolecules.

[8] Ding Q., Zhang Z., Wang C., Jiang J., Li G., Mai K. (2014). Non-isothermal crystallization kinetics and morphology of wollastonite-filled β-isotactic polypropylene composites. J. Therm. Anal. Calorim..

[9] Wang M., Lin L., Peng Q., Ou W., Li H. (2014). Crystallization and mechanical properties of isotactic polypropylene/calcium carbonate nanocomposites with a stratified distribution of calcium carbonate. J. Appl. Polym. Sci..

[10] Jiang C., Zhao S., Xin Z. (2015). Influence of a novel β-nucleating agent on the structure, mechanical properties, and crystallization behavior of isotactic polypropylene. J. Thermoplast. Compos. Mater..

[11] Cui L., Zhang Y., Zhang Y. (2006). Crystallization, melting behavior, and morphology of PP/Novolac blends. J. Polym. Sci. Part B Polym. Phys..

[12] Hu J.S., Kong B., Chao C.Y., Sun J. (2009). Study on liquid-crystalline copolymers as a new β-nucleator to induce crystallization structure of isotactic polypropylene. Chem. J. Chin. Univ..

[13] Hu J.S., Kong B., Sun J., Lv H.L., Li H. (2010). Effect of nematic liquid crystalline copolymer as a β-form nucleator on crystallization structure and thermal properties of isotactic polypropylene. Acta Polym. Sin..

[14] Sun J., Hu J.S., Chao C.Y., Guo Z.X., Qi Y. (2010). Influence of liquid crystalline copolysiloxane as a β-form nucleator on crystallization structure and morphology of isotactic polypropylene. Acta Chim. Sin..

[15] Sun J., Li Q., Yao X.J., Hu J.S., Qi Y. (2013). A nematic liquid crystalline polymer as highly active novel β-nucleating agent for isotactic polypropylene. J. Mater. Sci..

[16] Sun J., Hu J.S., Guo Z.X., Qi Y. (2013). Study on side-chain liquid–crystalline copolymer as a new β-nucleating agent to induce phase behavior of isotactic polypropylene. Colloid Polym. Sci..

[17] Yang R., Ding L., Chen W., Chen L., Zhang X., Li J. (2017). Chain folding in main-chain liquid crystalline polyester with strong π–π interaction: An efficient β-nucleating agent for isotactic polypropylene. Macromolecules.

[18] Yang R., Ding L., Chen W., Chen L., Zhang X., Li J. (2018). Nonisothermal crystallization, melting behaviors, and mechanical properties of isotactic polypropylene nucleated with a liquid crystalline polymer. Ind. Eng. Chem. Res..

[19] Su Z.Q., Dong M., Guo Z.X., Yu J. (2007). Study of polystyrene and acrylonitrile-styrene copolymer as special β-nucleating agents to induce the crystallization of isotactic polypropylene. Macromolecules.

[20] Su Z.Q., Chen X.N., Yu Z.Z., Zhang L. (2009). Morphological distribution of polymeric nucleating agents in injection-molded isotactic polypropylene plates and its influence on nucleating efficiency. J. Appl. Polym. Sci..

[21] Cao L., Dong M., Zhang A., Liu Y., Yang W., Su Z., Chen X. (2013). Morphologies and crystal structures of styrene-acrylonitrile/isotactic polypropylene ultrafine fibers fabricated by melt electrospinning. Polym. Eng. Sci..

[22] Phillips A.W., Zhu P.W., Edward G. (2010). Polystyrene as a versatile nucleating agent for polypropylene. Polymer.

[23] Phillips A.W., Bhatia A., Zhu P.W., Edward G. (2011). Shish formation and relaxation in sheared isotactic polypropylene containing nucleating particles. Macromolecules.

[24] Varga J., Karger-Kocsis J. (1996). Rules of supermolecular structure formation in sheared isotactic polypropylene melts. J. Polym. Sci. Part B Polym. Phys. Ed..

[25] Spina R., Spekowius M., Hopmann C. (2018). Simulation of crystallization of isotactic polypropylene with different shear regimes. Thermochim. Acta.

[26] Byelov D., Panine P., Remerie K., Biemond E., Alfonso G.C., de Jeu W.H. (2008). Crystallization under shear in isotactic polypropylene containing nucleators. Polymer.

[27] Deng Y., Mao X., Lin J., Chen Q. (2015). Compatibilization of polypropylene/poly (acrylonitrile-butadiene-styrene) blends by polypropylene-graft-cardanol. J. Appl. Polym. Sci..

[28] Lee H.G., Sung Y.T., Lee Y.K., Kim W.N., Yoon H.G., Lee H.S. (2009). Effects of PP-*g*-MAH on the mechanical, morphological and rheological properties of polypropylene and poly (acrylonitrile-butadiene-styrene) blends. Macromol. Res..

[29] Khare R.A., Bhattacharyya A.R., Kulkarni A.R. (2011). Melt-mixed polypropylene/acrylonitrile-butadiene-styrene blends with multiwall carbon nanotubes: Effect of compatibilizer and modifier on morphology and electrical conductivity. J. Appl. Polym. Sci..

[30] Luo Z., Lu Q., Ma F., Jiang Y. (2014). The effect of graft copolymers of maleic anhydride and epoxy resin on the mechanical properties and morphology of PP/ABS blends. J. Appl. Polym. Sci..

[31] Triantou M.I., Gavriel M., Sakellaris P., Tarantili P.A. (2016). The effect of compatibilizers and organically modified nanoclay on the morphology and performance properties of poly (acrylonitrile-butadiene-styrene)/polypropylene blends. Polym. Eng. Sci..

[32] Kum C.K., Sung Y.T., Kim Y.S., Lee H.G., Kim W.N., Lee H.S., Yoon H.G. (2007). Effects of compatibilizer on mechanical, morphological, and rheological properties of polypropylene/poly (acrylonitrile-butadiene-styrene) blends. Macromol. Res..

[33] Lee Y.K., Lee J.B., Park D.H., Kim W.N. (2013). Effects of accelerated aging and compatibilizers on the mechanical and morphological properties of polypropylene and poly (acrylonitrile-butadiene-styrene) blends. J. Appl. Polym. Sci..

[34] Sung Y.T., Kim Y.S., Lee Y.K., Kim W.N., Lee H.S., Sung J.Y., Yoon H.G. (2007). Effects of clay on the morphology of poly (acrylonitrile-butadiene-styrene) and polypropylene nanocomposites. Polym. Eng. Sci..

[35] Hom S., Bhattacharyya A.R., Khare R.A., Kulkarni A.R., Saroop M., Biswas A. (2009). PP/ABS blends with carbon black: Morphology and electrical properties. J. Appl. Polym. Sci..

[36] Khare R.A., Bhattacharyya A.R., Kulkarni A.R., Saroop M., Biswas A. (2008). Influence of multiwall carbon nanotubes on morphology and electrical conductivity of PP/ABS blends. J. Polym. Sci. Part B Polym. Phys..

[37] Shu Q., Zou X., Dai W., Fu Z. (2012). Formation of β-iPP in isotactic polypropylene/acrylonitrile–butadiene–styrene blends: Effect of resin type, phase composition, and thermal condition. J. Macromol. Sci. Part B.

[38] Varga J., Garzó G. (1990). The properties of polymer blends of the β-modification of polypropylene and an elastomer. Macromol. Mater. Eng..

[39] Grein C., Plummer C.J.G., Kausch H.H., Germain Y., Béguelin P. (2002). Influence of β nucleation on the mechanical properties of isotactic polypropylene and rubber modified isotactic polypropylene. Polymer.

[40] Jones A.T., Aizlewood J.M., Beckett D.R. (1964). Crystalline forms of isotactic polypropylene. Die Makromol. Chem..

[41] Zipper P., Janosi A., Wrentschur E. (1993). Scanning X-ray scattering of mouldings from semicrystalline polymers. J. Phys. IV.

[42] Liu J.R., Li C., Hu F.M. (2018). Effect of polystyrenes with different architectures on the β-nucleating efficiency and toughening of isotactic polypropylene. Polym. Int..

[43] Fujiyama M., Wakino T., Kawasaki Y. (1988). Structure of skin layer in injection-molded polypropylene. J. Appl. Polym. Sci..

